# Effects of Ion Combinations and Their Concentrations on Denitrification Performance and Gene Expressions of an Aerobic Strain Marinobacter Hydrocarbonoclasticus RAD-2

**DOI:** 10.3390/microorganisms11081867

**Published:** 2023-07-25

**Authors:** Junchi Li, Lei Cai, Huifeng Lu, Bin Ma, Guangsuo Chen, Dedong Kong, Yiming Hu, Ziran Ye, Yunjie Ruan

**Affiliations:** 1 Institute of Agricultural Bio-Environmental Engineering, College of Bio-Systems Engineering and Food Science, Zhejiang University, Hangzhou 310058, China; 22113045@zju.edu.cn (J.L.); 22213046@zju.edu.cn (Y.H.); 2 The Rural Development Academy, Zhejiang University, Hangzhou 310058, China; 0918314@zju.edu.cn; 3 Laboratory of Microbial Resources, College of Food Science and Biotechnology, Zhejiang Gongshang University, Hangzhou 310035, China; cailei@zjgsu.edu.cn; 4 Department of Environmental Engineering, College of Environmental and Resource Sciences, Zhejiang University, Hangzhou 310058, China; luhuifeng@zju.edu.cn; 5 Institute of Soil and Water Resources and Environmental Science, College of Environmental and Resource Sciences, Zhejiang University, Hangzhou 310058, China; bma@zju.edu.cn; 6 Institute of Digital Agriculture, Zhejiang Academy of Agricultural Sciences, Hangzhou 310021, China; ntzx@zju.edu.cn (D.K.); yezr@zaas.ac.cn (Z.Y.)

**Keywords:** aerobic denitrification, ion combinations, salinity, *Marinobacter hydrocarbonoclasticus* RAD-2, nitrogen-removal efficiency

## Abstract

Salinity is one of the most important factors affecting the nitrogen-removal efficiency of denitrifying bacteria. A series of different ion combinations and salinity gradients were carried out to clarify the effects of ion types and concentrations on nitrogen removal by halophilic aerobic denitrifying bacteria RAD-2. Nitrate concentrations, nitrite concentrations, TAN concentrations, and OD_600_ were monitored to investigate their effects on denitrification in each group. The results showed that Na^+^, K^+^, and Cl^-^ accelerated the denitrification process and improved nitrogen-removal efficiency at moderate additions, while Ca^2+^ and Mg^2+^ showed no significant effect. Na^+^ was effective alone, while K^+^ or Cl^-^ needed to be combined with at least one of Na^+^, K^+^, or Cl^-^ to achieve similar efficiency. The batch tests of salinity confirmed that the addition of a moderate concentration of NaCl/Na_2_SO_4_ could effectively improve nitrogen-removal efficiency, while excessive salinity might hinder denitrification metabolism. In the salinity range of 5~40‰, a 5‰ dosage might be the most economical method for strain RAD-2. Real-time PCR experiments on 17 key nitrogen metabolism-related genes revealed that chloride was widely involved in the nitrogen and carbon metabolism of microorganisms by altering cell osmotic pressure and opening ion channel proteins, thereby affecting the efficiency of denitrification. The results of this study may contribute to a better understanding of the different roles of various ions in aerobic denitrification and highlight the importance of salinity control in highly salted wastewater treatment.

## 1. Introduction

With the extensive use of feed in intensive aquaculture facilities, inorganic nitrogen pollution in aquatic ecosystems has caused serious environmental concerns, including eutrophication and poisoning of aquatic animals, which is mainly induced by nitrates [1]. Among various nitrate removal methods, biological heterotrophic denitrification is considered one of the most effective and economical ways of wastewater treatment due to its high flexibility and efficiency [2]. Since proposed by Robertson and Kuenen in 1984 [3], aerobic denitrification, conducted by aerobic denitrifiers, has been attracting more and more interest as one of the important nitrate metabolic pathways in biological denitrification [4]. Different from traditional anoxic nitrate removal, aerobic denitrifying microbes do not need a strictly anaerobic or anoxic environment for the denitrifying process because it is well known that they contain the napFDAGHBC gene cluster, which is insensitive to oxygen [5]. In the aerobic respiratory process conducted by aerobic denitrifiers, nitrate is converted gradually to dinitrogen (NO_3_^−^→NO_2_^−^→NO→N_2_O→N_2_) through nitrate reductase (Nar or Nao), nitrite reductase (Nir), nitric oxide reductase (Nor), and nitrous oxide reductase (Nos) encoded by the related genes napA, nirS, norB, and nosZ, respectively [4,6]. Conventional anoxic denitrification processes need spatial (e.g., anoxic/oxic process, A/O) or temporal (e.g., sequencing batch reactors, SBR) divisions to carry out complete nitrogen removal [7]. On the contrary, aerobic denitrifiers show wider niches and enhanced fitness to perform simultaneous nitrification and denitrification (SND) in one reactor due to their flexible metabolic pathways [8]. Therefore, these advantages can increase denitrification efficiency [9], simplify configurations and operations of reactors [10], and save land and operational costs [11] through the implication of aerobic bacteria in wastewater treatment.

It is well known that many parameters, such as oxygen level, temperature, pH, nitrate concentration, C/N ratio, salinity, and trace elements, affect the denitrification process [12,13]. At present, the research into aerobic denitrification is focused on the isolation of unique strains with excellent denitrification performance [14,15] and testing the effects of different operated parameters on nitrate removal of specific strains, such as dissolved oxygen (DO) concentration [16,17], C/N ratio [16,18], temperature [19,20,21], and pH [22,23]. Moreover, in order to make further efforts to clarify the mechanism of aerobic denitrification and make the aerobic denitrification process more economical and efficient, the kinetic affinity index [24,25] and the regulatory mechanisms of intracellular electron transfer [26] were characterized. Different antibiotics inhibition [8,27] and types of biofilm carriers [28,29] were also evaluated. However, the detailed mechanism and effective regulation have not been fully mastered, and the influence of ion species and concentrations is one of them. Though several proteins in the denitrification process require ions as a cofactor, the effects of ion compositions and concentrations on denitrification efficiency have been rarely presented in previous studies. Researchers observed that the activity of lipase produced by halotolerant *Bacillus* sp. VITL8 was enhanced in the presence of metal ions such as Ca^2+^, Mg^2+^, and Mn^2+^ [30]. And the plant growth regulating substances from halotolerant *Kosakonia sacchari* strain MSK1, which tolerated an unusually high salt concentration, increased with increasing NaCl concentration, illustrating that salinity stress induces the accumulation of stress biomarkers, antioxidant defense enzymes, and stressor metabolites [31]. Therefore, it is crucial to investigate the effect of ions on aerobic denitrification metabolism by aerobic denitrifiers, especially halophilic microbes. But to the best of our knowledge, none of the similar research has been conducted in previous studies.

In the present study, an aerobic denitrifying strain *Marinobacter hydrocarbonoclasticus* RAD-2 isolated from the previous study [32] was employed to investigate the effect of different ions on aerobic denitrification performance. Nitrate removal rate and bacterial biomass were used to determine whether the presence of ions contributes to the process of aerobic denitrification. In addition, the expression of a number of genes encoding related enzymes was characterized by real-time qPCR to illuminate the relevant metabolic mechanisms.

## 2. Materials and Methods

### 2.1. Characteristics of the Aerobic Denitrification Strain RAD-2

The aerobic denitrifying strain RAD-2 used in this study was isolated from a long-term poly (3-hydroxybutyrate-co-3-hydroxyvalerate)—(PHBV) supported denitrification reactor used for aquaculture effluent treatment. The reactor was operated under alternate aerobic/anoxic conditions [29], and the detailed isolation, screening, and identification information were in accordance with previous studies [32]. Identified as *Marinobacter hydrocarbonoclasticus* according to 16S rRNA-sequence phylogenetic analysis, strain RAD-2 was gram-negative, halotolerant, and bacilliform. And under aerobic conditions, its average nitrate- and nitrite-nitrogen-removal rates both reached 6 mg/(L·h). According to the gene annotation and the analysis of the Kyoto Encyclopedia of Genes and Genomes (KEGG), strain RAD-2 contained complete gene groups for the aerobic respiratory denitrification process, namely periplasmic dissimilatory nitrate reductase (NAP, napA), nitrite reductase (NIR, heme-containing cd1, nirS), nitric oxide reductase (cNOR, cytochrome c-dependent, norB), and N_2_O reductase (NOS, nosZ).

### 2.2. Effects of Different Combinations of Ions on Aerobic Denitrification Performance

Denitrification media (DM) with different ion combinations were prepared according to Table 1 to compare the effects of different ions on the aerobic denitrification efficiency of strain RAD-2. The total amount of carbon and nitrogen in each medium was identical. K_2_HPO_4_ was only added in the medium containing potassium ions, while in the Ca^2+^ + Mg^2+^ medium, glucose is added to ensure the absence of Na^+^ and K^+^ and to keep the amount of carbon constant. The formulation of LB is given in Table S1.

Subsequently, the seed suspension was inoculated at 30 °C in 250 mL Erlenmeyer flasks of DM at the ratio of 1:100 and shaken on a rotary shaker at 150 rpm. Samples were collected at 0 h, 24 h, and 48 h to determine the nitrate concentration, nitrite concentration, total ammonia nitrogen (TAN) concentration, and OD_600_.

### 2.3. Effect of Salinity on Aerobic Denitrification Efficiency of Strain RAD-2

In order to test the effect of salinity on the aerobic denitrification efficiency of strain RAD-2, the denitrification medium (DM) was prepared at different salinities (i.e., 0%, 5‰, 15‰, 25‰, and 40‰) according to Tables S2 and S3. Subsequently, the seed suspension of strain RAD-2 was inoculated at 30 °C in 250 mL Erlenmeyer flasks of DM at the ratio of 1:100, shaken on a rotary shaker at 150 rpm and sampled every 4 h to determine the nitrate concentration, nitrite concentration, TAN concentration, and OD_600_.

### 2.4. RT-qPCR Analysis

In order to further explore the mechanism of different types and concentrations of ions affecting the aerobic denitrification performance of strain RAD-2, real-time qPCR was performed to evaluate the expression of different key enzymes related to the denitrification process of strain RAD-2.

The basic NaCl/Na_2_SO_4_ denitrification medium for the qPCR assay was prepared according to Table S4. Seed suspensions were inoculated at 30 °C in 500 mL of each basic DM at the ratio of 1:200 and shaken on a rotary shaker at 150 rpm until the nitrate concentration dropped below 150 mg/L. Then 30 mL of the incubation was transferred to 100 mL of every corresponding DM with different concentrations for stimulation. Four hours later, samples were taken for the qPCR experiment. Three parallels were set for each concentration of NaCl/Na_2_SO_4_ in each medium and relevant results were standardized by 0.45 M of NaCl/Na_2_SO_4_. The housekeeping gene 16S ribosomal RNA was used as an internal control to normalize the data.

The qPCR amplification was performed with the following protocol: an initial denaturation step of 10 min at 95 °C, followed by 40 cycles of denaturation at 95 °C for 15 s, annealing at 60 °C (napA, nirS, and nosZ) or 56 °C (16S V3 region and norB) for 20 s, and a final extension at 72 °C for 15 s. All amplifications were conducted in triplicate using the SYBR Green Real-Time PCR Kit (Novland, Shanghai, China) and respective primers on an Mx3000P qPCR System (Agilent Technologies Co., Ltd., Beijing, China). Then nitrogen and carbon metabolic pathways were visualized with the Kyoto Encyclopedia of Genes and Genomes (KEGG) mapper (http://www.genome.jp/kegg/, accessed on 13 May 2022). The names and sequences of all relevant primers used for PCR amplification are listed in Table S5.

### 2.5. Analytical Methods

The solution samples were collected and filtered through a 0.45 μm filter membrane before water-quality analysis. Nitrate (NO_3_^−^-N), nitrite (NO_2_^−^-N), and TAN concentrations were analyzed according to standard methods [33]. Cell growth (OD_600_) was measured by using a spectrophotometer at 600 nm. All experiments were performed in multiple and data are shown as the mean ± SD.

Interventionary studies involving animals or humans, and other studies that require ethical approval, must list the authority that provided approval and the corresponding ethical approval code.

## 3. Results

### 3.1. Effects of Different Combinations of Ions on Aerobic Denitrification Performance

#### 3.1.1. Effectiveness Comparison for Different Combinations of Microelement Ions

**Figure 1:**
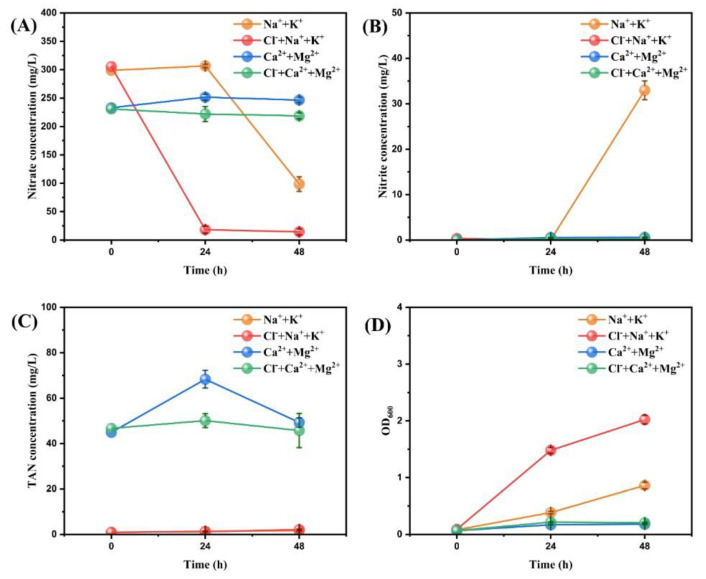
The changes of nitrate concentration (**A**), nitrite concentration (**B**), TAN concentration (**C**), and OD_600_ (**D**) after adding different concentrations of ion combinations to the medium of strain RAD-2. Data shown are mean ± SD (error bars) from three replicates.

In general, sodium, potassium, and chlorine are widely present in large amounts in water bodies. Calcium and magnesium ions are the main causes of hardness, which is a common problem in drinking water and industrial water [34]. Therefore, they are simply combined to illustrate the effect of different ion combinations on nitrogen removal. Using four simple ion combinations (Na^+^ + K^+^, Na^+^ + K^+^ + Cl^-^, Ca^2+^ + Mg^2+^, and Ca^2+^ + Mg^2+^ + Cl^-^), the effects of five microelement ions (Na^+^, K^+^, Ca^2+^, Mg^2+^, and Cl^-^) on aerobic denitrification were roughly clarified and relevant results were shown in Figure 1. Ion combination Ca^2+^ + Mg^2+^ had no effect on the aerobic denitrification process because nitrate concentration showed no drop after 48h. Nitrate in the Ca^2+^ + Mg^2+^ + Cl^-^ group was slightly reduced but negligible, showing the combination of Ca^2+^ + Mg^2+^ + Cl^-^ also had little effect. In contrast, Na^+^ + K^+^ group achieved a nitrate removal efficiency of 67.00 ± 3.49% at 48 h, although the nitrate content at 24 h was slightly higher than that at the initial time. In the Na^+^ + K^+^ + Cl^-^ group, 94.05 ± 1.58% nitrate was removed at 24 h and the nitrate content was further reduced at 48 h. The combinations of Na^+^ + K^+^ and Na^+^ + K^+^ + Cl^-^ both had positive effects on the aerobic denitrification process, whereas the combinations of Ca^2+^ + Mg^2+^ and Ca^2+^ + Mg^2+^ + Cl^-^ were not conducive to nitrogen removal.

In Figure 1B, nitrite in the Na^+^ + K^+^ group increased significantly at 48 h, while nitrite concentration in the other groups always remained at a very low level (<1 mg/L). In Figure 1C, the TAN concentration of the Na^+^ + K^+^ and Na^+^ + K^+^ + Cl^-^ groups was always around 2 mg/L, while that of the Ca^2+^ + Mg^2+^ and Ca^2+^ + Mg^2+^ + Cl^-^ groups was over 40 mg/L. In Figure 1D, the OD_600_ of the Ca^2+^ + Mg^2+^ and Ca^2+^ + Mg^2+^ + Cl^-^ groups did not increase significantly over time, while that of the Na^+^ + K^+^ group increased slightly at 24 h and climbed more significantly at 48 h. The OD_600_ value of the Na^+^ + K^+^ + Cl^-^ group was higher than that of the Na^+^ + K^+^ group with a very significant upward trend from 0 to 48 h.

#### 3.1.2. Further Discrimination for Different Effect of Na^+^, K^+^ and Cl^-^

**Figure 2:**
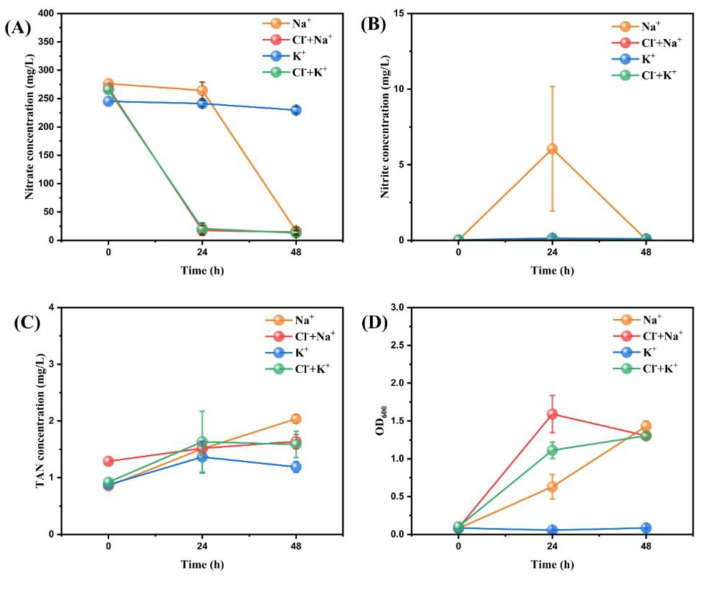
The changes of nitrate concentration (**A**), nitrite concentration (**B**), TAN concentration (**C**), and OD_600_ (**D**) after adding different concentrations of ion combinations to the medium of strain RAD-2. Data shown are mean ± SD (error bars) from three replicates.

Through the preliminary comparison of the five ions, Ca^2+^ and Mg^2+^ have been eliminated from the list of ions beneficial to aerobic denitrification. Next, Na^+^, K^+^, and Cl^-^ were further compared to determine their role in the nitrogen-removal process, and relevant results are shown in Figure 2.

Firstly, the variation of nitrate nitrogen within 48 h was shown in Figure 2A. Except for group K^+^, the other three groups showed similar nitrate removal efficiencies in 48 h (93.96 ± 1.95%, 94.62 ± 1.02%, 95.15 ± 1.21%, respectively), which implied that Na^+^, Na^+^ + Cl^-^, and K^+^ + Cl^-^ could promote effective nitrogen removal. In contrast, the nitrate removal efficiency of group K^+^ was only 6.28 ± 1.90%, indicating that K^+^ alone was detrimental to the aerobic denitrification process of strain RAD-2. In Figure 2B, the nitrite content in the Na^+^ group increased significantly at 24 h and then fell back to the initial level again at 48 h, while the other groups remained at the initial level. The TAN content in each group remained between 1 and 2 mg/L (Figure 2C), which is minimal when compared to the nitrite content. In Figure 2D, the three groups of Na^+^, Na^+^ + Cl^-^, and K^+^ + Cl^-^ all reached similar OD_600_ values at 48 h (1.4307, 1.3047, 1.3040, respectively) and their growth rates were as follows: Na^+^ + Cl^-^ > K^+^ + Cl^-^ > Na^+^. Meanwhile, group K^+^ showed no shift during the experimental period.

#### 3.1.3. The Threshold of Cl^-^ Concentration for Effective Nitrogen Removal

**Figure 3:**
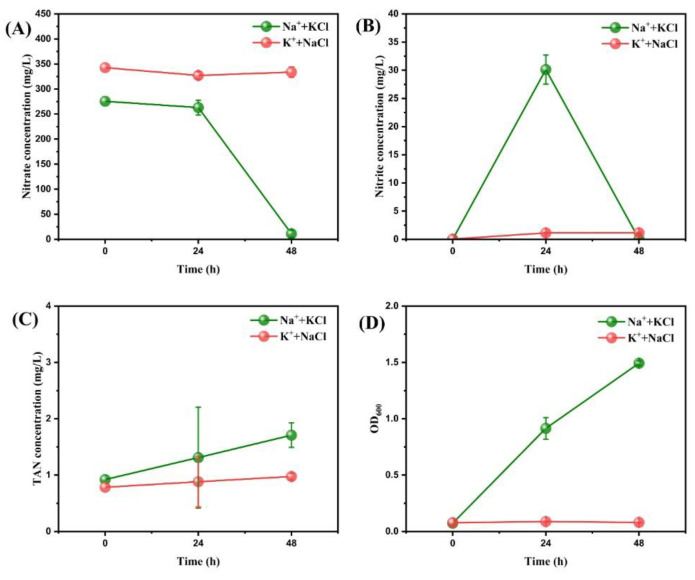
The changes of nitrate concentration (**A**), nitrite concentration (**B**), TAN concentration (**C**), and OD_600_ (**D**) after adding different concentrations of ions to the medium of strain RAD-2. Data shown are mean ± SD (error bars) from three replicates.

Though Cl^-^ played an important role in accelerating the aerobic denitrification process, the amount of Cl^-^ added in actual operation still needs further study due to economic considerations. Therefore, based on the formula of the Na^+^ group and K^+^ group in the previous experiment, we added 0.3 g of KCl and NaCl to determine the threshold of Cl^-^ concentration for effective nitrogen removal. Relevant results are shown in Figure 3. At 48 h, the nitrate removal efficiency of the Na^+^ + KCl group reached 95.97 ± 2.13%, slightly higher than that of the Na^+^ group, the Na^+^ + Cl^-^ group, and the Na^+^ + K^+^ + Cl^-^ group in the previous experiment. Nevertheless, the nitrate concentration did not decrease significantly at 24 h, while the nitrite concentration increased at the same time, showing an obvious plateau phenomenon. The results of the K^+^ + NaCl group were also similar to those of the K^+^ group in the previous experiment, but the reduction of nitrate nitrogen was only 2.60 ± 2.45% at 48 h, slightly lower than that of the K^+^ group.

### 3.2. Effect of Salinity on Aerobic Denitrification Efficiency of Strain RAD-2

**Figure 4:**
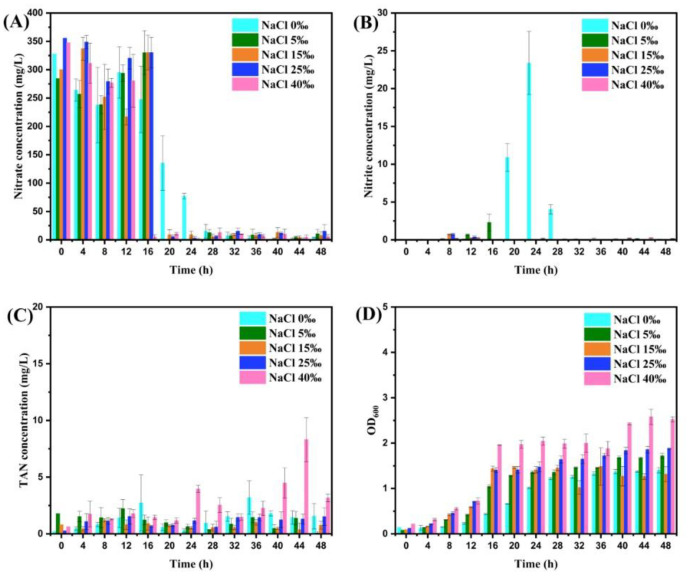
The changes of nitrate concentration (**A**), nitrite concentration (**B**), TAN concentration (**C**), and OD_600_ (**D**) every 4 h after adding different concentrations of NaCl to the medium of strain RAD-2. Data shown are mean ± SD (error bars) from three replicates.

**Figure 5:**
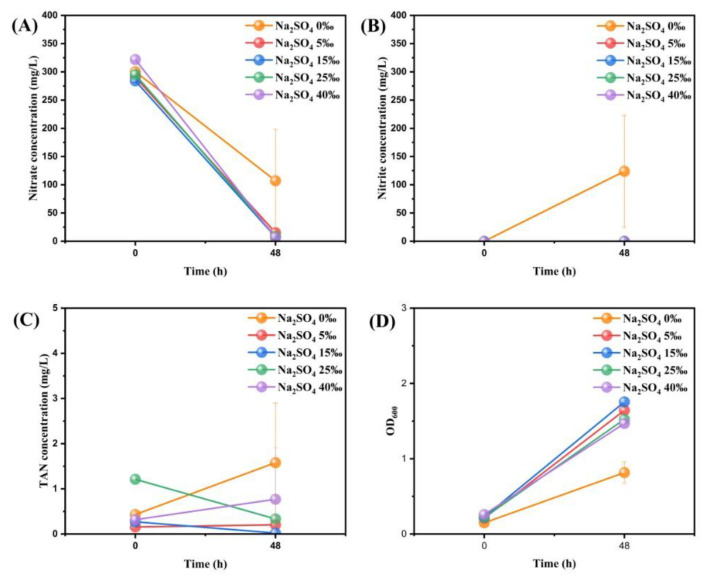
The changes of nitrate concentration (**A**), nitrite concentration (**B**), TAN concentration (**C**), and OD_600_ (**D**) after adding different concentrations of Na_2_SO_4_ to the medium of strain RAD-2. Data shown are mean ± SD (error bars) from three replicates.

In order to determine the most economical and effective concentration threshold of Na^+^ and Cl^-^, experiments were carried out with NaCl and Na_2_SO_4_ at different salinities and the experimental results are shown in Figure 4 and Figure 5. In Figure 4A, it is obvious that the nitrate concentration started to decrease significantly at 20 h under varying salinity. Although the nitrate removal efficiency of the NaCl 0‰ group was obviously lower than that of the other groups at about 24 h, it was not significantly different from that of the other groups at 48 h (*p* > 0.05). In Figure 4B, the nitrite content in the NaCl 0‰ group increased gradually and then decreased while the other groups did not have a similar situation, suggesting the existence of the platform period. In Figure 4C, the TAN content of each group did not exceed 5 mg/L; only the NaCl 40‰ group reached 8.31 ± 1.59 mg/L at 44 h, showing relatively obvious ammonia accumulation. In Figure 4D, the OD_600_ of each group showed a consistent trend of slow growth with time, and it was basically positively correlated with salinity at the same time.

Considering the important role of Na^+^ in the aerobic denitrification process, a similar salinity experiment was carried out after using Na_2_SO_4_ to eliminate the interference of Cl^-^ and the results were shown in Figure 5. It is obvious that the nitrate removal efficiency of the Na_2_SO_4_ 0‰ group was the lowest at 48h (only 64.33 ± 24.83%) while the other groups reached more than 90%. The Na_2_SO_4_ 0‰ group had similar nitrite accumulation to the Na^+^ group before at 48h and the growth rate of OD_600_ was also lower than that of the other groups. However, the data of the other four groups were highly similar and showed no significant difference.

### 3.3. Expression of Denitrifying Genes by RT-qPCR Analysis

**Figure 6:**
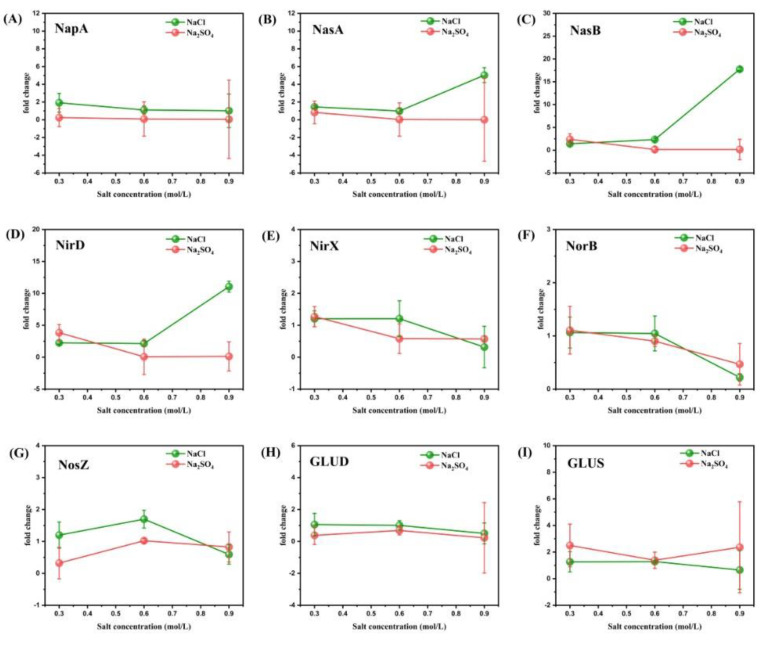
Fold change of nine enzymes (NapA, NasA, NasB, NirD, NirX, NorB, NosZ, GLUD, and GLUS) related to aerobic denitrification of strain RAD-2 with coculture of different concentrations of NaCl and Na_2_SO_4_. (drawn in subfigures (**A**–**I**), respectively) Fold change represents the expression of the experimental group relative to the benchmark, i.e., $2^{{-\Delta \Delta C}_{T}}$. Take the result when the salt concentration is 0.45 mol/L as the benchmark 1. The 16S ribosomal RNA was used as an internal control to normalize the data. Data shown are mean ± SD (error bars) from three replicates.

**Figure 7:**
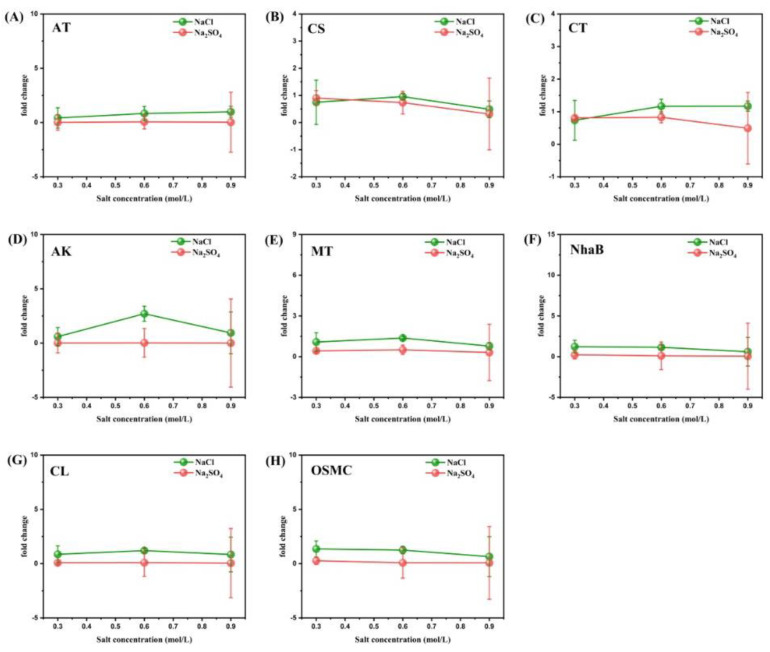
Fold change of eight genes (AT, CS, CT, AK, MT, NhaB, CL, and OSMC) related to carbon metabolism, ion transport, and osmotic pressure of strain RAD-2 with coculture of different concentrations of NaCl and Na_2_SO_4_ (drawn in subfigures (**A**–**H**), respectively) Fold change represents the expression of the experimental group relative to the benchmark, i.e., $2^{{-\Delta \Delta C}_{T}}$. Take the result when the salt concentration is 0.45 mol/L as the benchmark 1. The 16S ribosomal RNA was used as an internal control to normalize the data. Data shown are mean ± SD (error bars) from three replicates.

In order to study the specific mechanism behind the influence of different concentrations of Na^+^ and Cl^-^ on the denitrification efficiency of strain RAD-2, 17 key genes of strain RAD-2 were analyzed by real-time PCR and plotted in Figure 6 and Figure 7. The results showed that the gene expression stimulated by NaCl was higher than that stimulated by Na_2_SO_4_ at almost all salt concentrations, suggesting that Cl^-^ played a more advantageous role than Na^+^ in the denitrification of strain RAD-2. In addition, the increase in salinity did not necessarily lead to better denitrification as we would expect since excessive salinity might be detrimental to nitrogen removal.

## 4. Discussion

### 4.1. Effects of Different Combinations of Ions on Aerobic Denitrification Performance

#### 4.1.1. Effectiveness Comparison for Different Combinations of Microelement Ions

It is obvious that both Ca^2+^ + Mg^2+^ and Ca^2+^ + Mg^2+^ + Cl^-^ groups had no effect on the nitrogen removal of aerobic denitrifying bacteria RAD-2. Within 48 h, the TAN content was at a high level and the nitrate concentration did not decrease significantly in these two groups. In addition, the OD_600_ did not increase significantly reflecting that the number of cells remained constant. These phenomena consistently indicated that the presence of Ca^2+^ and Mg^2+^ might have a blocking effect on the aerobic denitrification process. Previous studies have shown that some heavy metal ions (e.g., Cd^2+^, Cu^2+^, Ni^2+^, Zn^2+^, Cr^6+^, Fe^2+^, Pb^2+^) have different inhibitory effects on the metabolic processes of aerobic denitrifying strains [35]. Their effects vary greatly depending on the type, valence, and concentration [36]. Calcium channels in bacteria can cause the accumulation of Ca^2+^ around cell walls, triggering nucleation and crystallization [37,38,39,40]. The formation of precipitates such as calcium carbonate may be the direct cause of the weakening of aerobic denitrification metabolism of strain RAD-2. Our experimental results were in line with the biomineralization phenomenon.

On the other hand, Na^+^ + K^+^ and Na^+^ + K^+^ + Cl^-^ groups both achieved a significant reduction of nitrate and a visible boost in the number of cells, while maintaining their TAN concentrations at very low levels within 48 h. This suggests that these ions are likely to have a positive effect on the growth, reproduction, and nitrogen-removal metabolism of strain RAD-2. Santo Fabio Corsino et al. [41] successfully sustained simultaneous nitrification–denitrification at salinities (NaCl) up to 50 g L^−1^ in the treatment of fish canning wastewater by aerobic granular sludge. A maximum total nitrogen (TN) removal efficiency of 98% was achieved at 50 g NaCl L^−1^ and its carbon removal efficiency was over 90%. This is consistent with the conclusions we obtained.

Furthermore, the main difference between the two groups is that the nitrate removal efficiency of the Na^+^ + K^+^ + Cl^-^ group was 94% at 24 h, while only 67% was achieved in the Na^+^ + K^+^ group at 48 h. In addition, the nitrite content of the Na^+^ + K^+^ group increased significantly at 48h, whereas the Na^+^ + K^+^ + Cl^-^ group remained constant (Figure 1B). These phenomena indicate that the addition of chloride probably accelerated the process of denitrification. Specifically, it might reduce the accumulation of intermediate products and shorten the lag time of the NO_3_^−^→NO_2_^−^→NO_x_ process.

#### 4.1.2. Further Discrimination for Different Effect of Na^+^, K^+^ and Cl^-^

Apparently, Na^+^ is the most important of the three ions in the aerobic denitrification process of strain RAD-2. Na^+^ alone had a similar effect on the nitrate removal efficiency as the ion combinations such as Na^+^ + Cl^-^ and K^+^ + Cl^-^. This indicates that Na^+^ itself plays an excellent role in promoting the denitrification process of strain RAD-2. The roles of K^+^ and Cl^-^ are less strong than that of Na^+^. Group K^+^ only achieved a small amount of nitrate reduction, which was significantly lower than the other groups in efficiency. Only when combined with Na^+^ or Cl^-^ could the ideal nitrate removal efficiency be achieved within 48 h. The combination of Cl^-^, Ca^2+^, and Mg^2+^ could not achieve effective nitrogen removal, except when Cl^-^ was combined with Na^+^ or K^+^, which even exceeded the Na^+^ group. This result indicates that Cl^-^ could be a "catalyst" that can enhance the effect of Na^+^ and K^+^ on nitrogen removal, but the mechanism involved needs to be further investigated.

#### 4.1.3. The Threshold of Cl^-^ Concentration for Effective Nitrogen Removal

Considering the results of previous experiments, both the Na^+^ + KCl group and the Na^+^ group showed an obvious plateau period, which was not observed in the Na^+^ + Cl^-^ group, the K^+^ + Cl^-^ group, and the Na^+^ + K^+^ + Cl^-^ group. It indicated that 0.3 g/L KCl is insufficient to achieve the goal of accelerating nitrogen removal. On the other hand, the K^+^ + NaCl group was similar to the K^+^ group and did not achieve the ideal nitrogen-removal efficiency, while the K^+^ + Cl^-^ group achieved an excellent nitrate removal efficiency of 95.15 ± 1.21%. Therefore, it can be concluded that >0.3 g/L NaCl is needed in order to improve the efficiency of nitrogen removal.

### 4.2. Effect of Salinity on Aerobic Denitrification Efficiency of Strain RAD-2

It reaffirmed that the addition of NaCl with appropriate content could indeed accelerate the aerobic denitrification process and reduce the platform period time. Wang et al. [42] observed the highest removal percentages of NO_2_^−^–N (99%) and NO_3_^−^–N (95%) by strain *Pannonibacter phragmitetus* F_1_ at NaCl concentration of 10 g/L. In addition, strain *Pannonibacter phragmitetus* F_1_ can adapt to a wide range of neutral and alkaline environments (pH of 7–10) and show high tolerance of NaNO_2_ concentration (0.4–1.6 g/L) when the NaCl concentration was 10 g/L. Chen et al. [43] found that organic matter and nitrogen removal can be achieved simultaneously in a single-stage pressurized biofilm reactor under a salinity of up to 3.0%, achieving TN removal efficiency via an SND of up to 98%. Luo et al. [44] established an efficient SND-MBR high salinity system for tungsten smelting wastewater and the TN removal efficiency reached 95.55% after stable operation at 3.0% salinity. These findings consistently indicate that moderate amounts of NaCl addition can effectively improve nitrogen-removal efficiency and do not inhibit denitrification.

However, it is worth noting that its promoting effect had little relationship with salinity when the amount of NaCl added was between 5~40‰. Although the OD_600_ results showed that high salinity had a positive effect on the proliferation of the halophilic microorganism, it would lead to the accumulation of TAN to a certain extent and increase the economic burden when the salinity reached 40‰. High salinity may decrease cell activity and even cause cell plasmolysis in a biological system for wastewater treatment [43]. A possible explanation is that high salinity leads to the over-proliferation of halophilic microorganisms and competition for limited nutrients, eventually resulting in the death of bacteria and the dissolution of organic nitrogen from the cell in the form of ammonia nitrogen. Several studies [44,45,46,47] have confirmed that excessive salinity alters osmotic pressure and inhibits the activity of associated denitrifying enzymes and electron transport systems, thereby negatively affecting nitrogen removal. Chen et al. [48] demonstrated that NaCl, KCl, and Na_2_SO_4_ exerted different degrees of inhibition on HN-AD bacteria *Acinetobacter* sp. TAC-1 by batch experiments, with half concentration inhibition constant values of 0.205, 0.238, and 0.110 M, respectively. Different bacteria have different levels of tolerance to salinity, so 5‰ may be the most economical salinity of strain RAD-2 to effectively promote the aerobic denitrification process.

It can be assumed that varied salinity of Na_2_SO_4_ within 5~40‰ had a similar effect on the aerobic denitrification process of strain RAD-2. It can be seen that 5‰ may be the most economical salinity to effectively promote the aerobic denitrification process, which is consistent with the previous NaCl experiment.

### 4.3. Expression of Denitrifying Genes by RT-qPCR Analysis

According to the KEGG Pathway Database, various enzymes are encoded by corresponding functional genes involved in the nitrogen metabolism of the strain RAD-2 as shown in Figure 6. As one of the most critical enzymes in the denitrification metabolic pathway, periplasmic dissimilatory nitrate reductase (NapA, EC 1.9.6.1) had a higher abundance in the NaCl medium than in the Na_2_SO_4_ medium for all different concentrations. It indicated that Cl^-^ could effectively promote the synthesis of nitrate reductase and thus accelerate the process of dissimilatory nitrate reduction. It is worth noting that this experiment was based on an overall high level of salinity because 0.3M NaCl is equivalent to 17.5‰ salinity and 0.3M Na_2_SO_4_ is more than 40‰ salinity. The expression of nitrate reductase decreased slowly with increasing salinity, which is consistent with the conclusion that denitrification metabolism was not more active at salinities over 5‰ and excessive salinity would lead to a decrease in nitrogen-removal efficiency.

As for assimilatory nitrate reduction, the assimilatory nitrate reductase (NasA, EC 1.7.99.-) and the corresponding electron transfer subunit (NasB, EC 1.7.99.-) showed a similar trend in Figure 6C. The expression of two genes in the NaCl group was higher than that in the Na_2_SO_4_ group at almost all salinities, indicating that Cl^-^ stimulates the assimilation nitrate reduction pathway. Moreover, the assimilation nitrate reduction metabolism in the NaCl group increased significantly with increasing salinity, especially at high salinity (0.9 M). However, the initially inactive expression in the Na_2_SO_4_ group gradually decreased with the rise of salinity, showing that assimilation nitrate metabolism is not sensitive to Na^+^.

Still, the expression trends of nitrite reductase large subunit (NirD, EC 1.7.1.15) and nitrate reductase (NirX, EC 1.7.1.1 1.7.1.2 1.7.1.3) were different in the two groups. At a concentration of 0.3M, the expression of both NirD and NirX was lower in the NaCl group than in the Na_2_SO_4_ group. The opposite was true at 0.6M concentration. When the salinity reached 0.9M, the expression of NirD was much higher in the NaCl group than that in the Na_2_SO_4_ group, while the expression of NirX was slightly lower. This reflected the complex change in the nitrite reduction metabolism process with increasing salinity. In general, the addition of Cl^-^ at higher salinity was more conducive to the nitrite reduction process. For the Na_2_SO_4_ group, the expression of NirD and NirX decreased with increasing salinity, which again confirmed our previous conclusion. For the NaCl group, the expression of NirD was positively correlated with salinity, while NirX was negatively correlated.

For nitric oxide reductase (NorB, EC 1.7.2.5), the NaCl group and the Na_2_SO_4_ group showed the same trend of change, i.e., the expression decreased with increasing salinity. It is also consistent with our previous conclusion that excessive salinity would lead to a decrease in nitrogen-removal efficiency. The expression of genes encoding nitrous-oxide reductase (NosZ, EC 1.7.2.4) showed a novel trend. With increasing salinity, the expression of genes in both the NaCl group and the Na_2_SO_4_ group first increased and then decreased, suggesting that a certain salinity between 0.3 M and 0.9 M might be the most suitable for nitrous oxide reduction metabolism. Moreover, the expression of NosZ in the NaCl group was always higher than that in the Na_2_SO_4_ group at 0.3M and 0.6M, which also showed that the presence of Cl^-^ had a positive effect on the nitrous oxide reduction metabolism.

NAD-glutamate dehydrogenase (GLUD, EC 1.4.1.3) and glutamate synthase large subunit (GLUS, EC 6.1.1.24) are usually responsible for the fixation of ammonia to glutamic acid, reflecting the biomass of microbes [49]. In general, the expression changes of these two enzymes were relatively complex, and their expression activity decreased slowly with increasing NaCl concentration. This is in line with the common sense that the increase of osmotic pressure leads to the death of microorganisms. In addition, it also confirms our previous inference that the biomass will decrease with increasing salinity within a certain range. GLUD expression in the NaCl group was higher than that in the Na_2_SO_4_ group at all concentrations while GLUS expression was the opposite, reflecting the complexity of ammonia nitrogen synthesis.

The presence of Na^+^ and Cl^-^ can not only alter the expression of denitrification-related genes but also affect other metabolic processes of strain RAD-2. As one of the most important metabolic pathways of microorganisms, we selected five key genes closely related to carbon metabolism for the qPCR experiment. They are encoding for phosphate acetyltransferase (AT, EC 2.3.1.8), citrate synthase (CS, EC 2.3.3.1), acetyl-CoA/propionyl-CoA carboxylase carboxyl transferase subunit (CT, EC 6.4.1.2 6.4.1.3 2.1.3.15), acetate kinase (AK, EC 2.7.2.1), and 2S-malonyltransferase (MT, EC 2.3.1.39). Although their expression trends with salinity varied, the enzyme activity of the Na_2_SO_4_ group was always very low. In addition, the expression level of genes in the NaCl group was often higher than that in the Na_2_SO_4_ group, indicating that the presence of Cl^-^ can effectively benefit microbial carbon metabolism, proliferation, energy production, and transformation processes. Especially for AK, the expression of the NaCl group was significantly higher than that of the Na_2_SO_4_ group at 0.6M, illustrating that Cl^-^ may intervene in the process of the tricarboxylic acid cycle by accelerating the synthesis and conversion of acetyl-CoA, thus improving the carbon metabolism level of microorganisms. In addition, the results of AT, CS, CT, and MT showed that Cl^-^ also had different effects on the biosynthesis and transfer of citrate and fatty acid.

Sodium/proton antiporter (NhaB) [50,51], chloride channel protein (CL) [52], and osmotically inducible peroxiredoxin (OSMC) [53] were also amplified by qPCR. The results were highly consistent: the activity of three proteins was higher in the NaCl group than in the Na_2_SO_4_ group at different salinities. This partly explains the mechanism of the extensive involvement of chloride in the nitrogen and carbon metabolism of strain RAD-2. By changing the osmotic pressure, the sodium/proton antiporter and chloride protein channels were activated to a greater extent, thus activating the entire microbial metabolism. It is worth noting that the activity of three proteins in both groups slowly decreased with increasing salinity, which further explains the phenomenon we observed earlier. The ionic protein channel slowly closed at excessive salinity, leading to the reduction of transport speed and metabolic activity, and no longer increasing the nitrogen-removal efficiency.

## 5. Conclusions

A variety of different ion combinations were applied to the medium of the halophilic aerobic denitrifying strain RAD-2 to confirm that the presence of ions does influence denitrification. Among the five ions involved in the experiment, Na^+^, K^+^, and Cl^-^ were beneficial to nitrogen removal, while Ca^2+^ and Mg^2+^ did not have a significant effect. Na^+^ was the most effective ion, as its presence alone in the batch test achieved similar nitrate removal efficiency as the combination of the three ions. K^+^ and Cl^-^ must be combined with at least one of Na^+^, K^+^, and Cl^-^ to achieve similar efficiency. Adequate amounts of chloride ions provided excellent support for K^+^/Na^+^ to accelerate the denitrification process and improve nitrate-removal efficiency. This was confirmed at the genetic level in qPCR experiments that Cl^-^ was widely involved in the nitrogen and carbon metabolism of microorganisms by altering cell osmotic pressure and opening ion channel proteins. Moderate amounts of NaCl/Na_2_SO_4_ addition could effectively improve nitrogen-removal efficiency, while excessive salinity might hinder denitrification metabolism. In the salinity range of 5~40‰, a 5‰ dosage may be the most economical concentration for strain RAD-2.

## Figures and Tables

**Table 1:** Formulations of DM with different ion combinations.

DM	Na^+^	Na^+^ + Cl^-^	K^+^	K^+^ + Cl^-^	Na^+^ + K^+^	Na^+^ + K^+^ + Cl^-^	Ca^2+^ + Mg^2+^	Ca^2+^ + Mg^2+^ + Cl^-^	Na^+^ + KCl	K^+^ + NaCl
NaNO_3_ (g)	1.821	1.821	-	-	0.911	0.911	-	-	1.821	-
KCl (g)	-	-	-	-	-	-	-	-	0.3	-
KNO_3_ (g)	-	-	2.166	2.166	1.083	1.083	-	-	-	2.166
NaCl (g)	-	25	-	25	-	25	-	25	-	0.3
Ca(NO_3_)_2_ (g)	-	-	-	-	-	-	1.758	1.758	-	-
MgCl_2_ (g)	-	-	-	-	-	-	0.3	0.3	-	-
MgSO_4_·7H_2_O (g)	0.2									
NaAc (g)	10	10	-	-	10	10	-	-	10	-
KAc (g)	-	-	11.964	11.964	-	-	-	-	-	11.964
Glucose (g)	-	-	-	-	-	-	7.321	7.321	-	-
K_2_HPO_4_ (g)	-	-	1.0	1.0	1.0	1.0	-	-	1.0	1.0

Notes: All chemicals shown above were of analytical grade and were used without further purification. The initial pH of all media was set to 7.2, and all media were autoclaved for 20 min at 121 °C. Set three parallel for each medium.

## Data Availability

No new data were created.

## Supplementary Materials

The following supporting information can be downloaded at: https://www.mdpi.com/article/10.3390/microorganisms11081867/s1, Table S1: The recipe of LB; Table S2: Formulation of DM to test the effect of Cl^-^/Na^+^; Table S3: The composition of the trace-element solution; Table S4: Formulation of basic NaCl/Na_2_SO_4_ DM for qPCR; Table S5: Names and sequences of primers for qPCR amplification.
