# Supplementary material for: Effects of Ion Combinations and Their Concentrations on Denitrification Performance and Gene Expressions of an Aerobic Strain Marinobacter Hydrocarbonoclasticus RAD-2
Source: Microorganisms. 2023 Jul 25;11(8):1867. doi: 10.3390/microorganisms11081867 (PMC10456938; doi:10.3390/microorganisms11081867)
Supplement: Supplementary file 1 [file microorganisms-11-01867-s001.zip › microorganisms-2398471-supplementary.pdf]

Supplementary materials

Table S1 The recipe of LB

Materials	Weight(g)
Tryptone	10
Yeast Extract	5
NaCl	10

Notes: Dissolve components in 1 L of distilled water and adjust pH to 7.2.

Table S2 Formulation of DM to test the effect of Cl^-/Na^+

Materials	Quantity
KNO_3	2.166g
$MgSO_4 \cdot 7H_2O$	0.2g
K_2HPO_4	1.0g
NaAc	10g
the trace-element solution	10mL
NaCl/Na_2SO_4(0‰, 5‰, 15‰, 25‰, 40‰)	0g, 5g, 15g, 25g, 40g

Notes: All these materials are dissolved in 1 L of distilled water and set three parallel for each concentration of NaCl/Na_2SO_4. The initial pH of all media was set to 7.2, and all media were autoclaved for 20 minutes at 121 °C.

Table S3 The composition of the trace-element solution

Materials	Weight(g)
EDTA	50
$ZnSO_4 \cdot 7H_2O$	3.92
$CaCl_2$	5.5
$MnCl_2$	3.22
$FeSO_4 \cdot 7H_2O$	5.0
$(NH_4)_6Mo_7O_2$	1.1
$CuSO_4 \cdot 5H_2O$	1.57
$CoCl_2 \cdot 6H_2O$	1.61

Notes: All these materials are dissolved in 1 L of distilled water and adjust pH to 7.2.

Table S4 Formulation of basic NaCl/Na_2SO_4 DM for qPCR

Materials	Quantity
KNO_3	2.166g
$MgSO_4 \cdot 7H_2O$	0.2g
KAc	11.964g
the trace-element solution	10mL
NaCl/Na_2SO_4	0.3M, 0.6M, 0.9M

Notes: All these materials are dissolved in 1 L of distilled water. The initial pH of all media was set to 7.2, and all media were autoclaved for 20 minutes at 121 °C.

Table S5 Names and sequences of primers for qPCR amplification

Abbreviation	Name	Sequence (5'→3')
16S rRNA	16S ribosomal RNA	CCTACGGGAGGCAGCAG<br>ATTACCGCGGCTGCTGG
NapA	periplasmic dissimilatory nitrate reductase	GCAACGTAGCCAAGTACAG<br>TTCGCAAGAGCAGATCCAG
NasA	assimilatory nitrate reductase	AACGCCATCATTAACTGCC<br>TGCCAGAAATCCGAAACCC
NasB	assimilatory nitrate reductase (electron transfer subunit)	TGATTACCCACGATGTGGAC<br>ACAGGAACGACAACACCTC
NirD	nitrite reductase large subunit	TTACCCTGGAGAACCGCTAC<br>GCCATTGCCACACACATAC
NirX	nitrite reductase	TGCCAAGATTGTTCGCTCC<br>GCCGTAACTGATAAACGCC
NorB	nitric oxide reductase	GCAACCCACGATAACCAAG<br>GAAGGAGAACAGCCACAATAC
NosZ	nitrous-oxide reductase	GGTGAAGGAGGGTGATGAAG<br>GGAGAGCATGACAGAACCAG
GLUD	NAD-glutamate dehydrogenase	CCTTCAACCACATCCACATC<br>CCCAGCAGCTTTTTCATTTC
GLUS	glutamate synthase large subunit	CAGGAATATCACGCCTTCAAC<br>ATTGACTGGATGCCCTCAC

AT	2acetyltransferase _F	AACCGAAGCCTATATCGCC
	2acetyltransferase _R	CCCACCTTGTTAAGAATGACC
CS	2citrate synthase_F	CCCAGCGAAGAAGAAAACAAG
	2citrate synthase_R	CGTGATAGAACGCAGACAG
CT	2carboxyl transferase_F	ATCACCGACGAAATCAGCC
	2carboxyl transferase_R	ATGCAGCTCGTCAAAATCC
AK	2acetate kinase_F	GCTTACCGATACGCACTACC
	2acetate kinase_R	TGATGCTACAACCGTTACCC
MT	2S-malonyltransferase_F	GCTCAAGCAGCTATACTCAC
	2S-malonyltransferase_R	TCAGCCAGAGAATCAGGAC
NhaB	sodium/proton antiporter	GCAACTTCTTCTACAACGACC
		GCAGTAGTCATCCAGAATGCC
CL	chloride channel	AATGGCCACTGGAGCATTTC
	protein(Marinbacter)	ATGAACGATGCCGACTTTGC
OSMC	osmotically inducible	GCAACTTCTTCTACAACGACC
	peroxiredoxin	GCAGTAGTCATCCAGAATGCC
